# London in 1828

**Published:** 1889-03-30

**Authors:** 


### LONDON IN 1828.

"I looked on the raging street, where a parti-coloured coil of men, women, and children, horses, stage-coaches, and with them a funeral, whirled groaning and creeping along, and it seemed to me as though all London were a Beresina Bridge, where every one presses on in mad haste to save his scrap of life, where the daring rider stamps down the poor pedestrian, where every one who falls is lost for ever; where the best friends rush, without feeling, over each other's corpses, and where thousands, weak and bleeding, grasp in vain at the planks of the bridge, and slide down into the ice-pit of death."—*Heine.*

